# Supplementary material for: A novel nairovirus associated with acute febrile illness in Hokkaido, Japan
Source: Nat Commun. 2021 Sep 20;12:5539. doi: 10.1038/s41467-021-25857-0 (PMC8452618; doi:10.1038/s41467-021-25857-0)
Supplement: Supplementary file 1 — Supplementary Information [file 41467_2021_25857_MOESM1_ESM.pdf]

Supplementary Information

A novel nairovirus associated with acute febrile illness in Hokkaido, Japan

Supplementary Tables 1
Supplementary Figures 7

Supplementary Tables

Supplementary Table 1. Ferritin levels in blood of two patients infected with YEZV

Day after fever onset	4	5	8	11	12	15	19	21
Patient 1	NA	55,200	107,800	NA	4,254	1,782	1,149	NA
Patient 2	3,403.1	8,577	886	388	NA	251	NA	207

NA: Not available, unit: ng/ml

Supplementary Table 2. Sequences used in the phylogenetic analyses

Virus	Strain/isolate	Accession number		
		L segment	M segment	S segment
Yezo virus	HH007-2016	NA*	NA	LC628643
	HH008-2017	NA	NA	LC628644
	HH009-2017	NA	NA	LC628645
	HH001-2019	LC621352	LC621353	LC621354
	HH011-2020	LC621358	LC621359	LC621360
	HH003-2020	LC621355	LC621356	LC621357
Artashat virus	LEIV-10898Az	NC_043440	NC_043442	NC_043441
Burana virus	760	NC_043439	NC_043438	NC_043437
Caspiy virus	LEIV-63Az	KP792708	KP792709	KP792710
Chim virus	LEIV-858Uz	NC_043434	NC_043436	NC_043435
Clo Mor virus	ScotAr7	NC_034561	NC_034554	NC_034562
Crimean-Congo hemorrhagic fever virus	IbAr10200	NC_005301	NC_005300	NC_005302
Dera Ghazi Khan virus	JD254	NC_034520	NC_034510	NC_034521
Dugbe virus	ArD 44313	NC_004159	NC_004158	NC_004157
Estero Real virus	K 329	MK896579	MK896578	MK896577
Farallon virus	CalAr846	NC_034502	NC_034494	NC_034503
Geran virus	LEIV-10899Az	KP792714	KP792715	KP792716
Gossas virus	DakAnD 401	KR534878	KR534877	KR534876
Hazara virus	JC280	NC_038709	NC_038710	NC_038711
Huangpi Tick virus 1	H124-1	NC_031135	NC_031136	NC_031137
Issyk-Kul virus	LEIV-315K	KR709221	KR709220	KR709219
Kasokero virus	Z-52963	NC_036636	NC_029933	NC_029932
Keterrah virus	P61361	NC_034392	NC_034388	NC_034389
Leopards Hill virus	11SB17	NC_025831	NC_025832	NC_025833
Meram virus	M1	MN972594	MN972595	MN972596
Nairobi sheep disease virus	Jilin	NC_034387	NC_034391	NC_034386
Pacific coast tick nairovirus	Docc2011cons	KU933933	KU933934	KU933935
Paramushir virus	LEIV-1149K	KP792717	KP792718	KP792719
Qalyub virus	ErAg370	NC_034511	NC_034522	NC_034512
Sanxia Water Strider virus 1	SXSSP08	NC_031141	NC_031142	NC_031143

Virus	Strain/isolate	Accession number		
		L segment	M segment	S segment
Saphire II virus	RML 52323-14	KU925485	KU925486	KU925487
Shayang Spider virus 1	SYZZ-4	NC_031220	NC_031218	NC_031219
Songling virus	HLJ1202	MT328776	MT328775	MT328777
Sulina virus	IxriSL16-01	MT263282	MT263283	MT263284
Tacheng Tick virus 1	TC253	NC_031284	NC_031285	NC_031286
Tamdy orthonairovirus	TT1	MN792651	MN792652	MN792653
Thiafora virus	AnD 11411	NC_039220	NC_039221	NC_039222
Tofla virus	Toku Hfla 2013	NC_029124	NC_029123	NC_029122
Uzun Agach virus	LEIV-Kaz155	KP792741	KP792742	KP792743
Vinegar Hill virus	CS1499	MF176881	MF176882	MF176883
Wenzhou Tick virus	TS1-2	NC_031291	NC_031288	NC_031289
Xinzhou Spider virus	XZZZ-2	KM817702	KM817729	KM817762
Yogue virus	DakAnD 56	NC_029931	NC_029935	NC_029936

[*]Not analyzed.

Supplementary Table 3. Primers used for RT-PCR and Sanger sequencing

Primer name	Target segment	Direction	Sequence (5' -> 3')
YEZV_L-22F	L	Forward	TCCCTACACTGAATCAAGCAAGAA
YEZV_L-271R	L	Reverse	TGGCCACAAACAAGTTATTGATCC
YEZV_L-550F	L	Forward	CCCATTATAATCTGGAGGGCAGAA
YEZV_L-649R	L	Reverse	GCGATTGAATCAGGTTTATCTCTCC
YEZV_L-978F	L	Forward	CTGCAATGTGTCAGTCCTTTTCAT
YEZV_L-1262R	L	Reverse	AGGTGGAAAGTAGTATGACTGTGG
YEZV_L-1670F	L	Forward	AGGCTTCAGACAAGGAGTCT
YEZV_L-1958R	L	Reverse	TCCAACTCAAGCTGTCCAAC
YEZV_L-2434F	L	Forward	AGAAGAACCAAGGAAAAGGCATTT
YEZV_L-2480R	L	Reverse	AGCGATCAAGACTCCTCAATTCTT
YEZV_L-2766F	L	Forward	CTCACCAGAAGATGCAAGACTACT
YEZV_L-2809R	L	Reverse	GGAAGCTGATCGACTCTTTGAGTA
YEZV_L-2950F	L	Forward	GCCTGGAATGCGAAACTAGA
YEZV_L-3126F	L	Forward	AGTGGAAGAAACCAAAAACAAGCA
YEZV_L-3208R	L	Reverse	CCTCCATCAGCCAACCCAAA
YEZV_L-4146R	L	Reverse	TTCCTGGAACCAGTCAAAGC
YEZV_L-4635F	L	Forward	CCTCTCAGTTGTGTTTGGGC
YEZV_L-4816R	L	Reverse	TGGATACCCCGGACTCTATC
YEZV_L-5842R	L	Reverse	TCCAGTTGTAGAAAGAAAGCTTGC
YEZV_L-5912F	L	Forward	TGAAAAGTATGGCTTCAGGAGTGA
YEZV_L-6310R	L	Reverse	TAAGAGCAATTTCTTCCCTGGTGA
YEZV_L-6491F	L	Forward	GGCTTACTTCTCTTGGACCTGTTA
YEZV_L-7104F	L	Forward	CAATAGCCATCTCAAGGAAGCAAT
YEZV_L-7487F	L	Forward	AGTCAATTGAGATCTGGAGTGACC
YEZV_L-7772R	L	Reverse	AAAGTGCTCTTACTTATTGCTCCG
YEZV_L-8365F	L	Forward	GACAAGCTAACTTCAGGAGAGTCA
YEZV_L-8832F	L	Forward	AAGGCTATCCATAAACGAGCTGAT
YEZV_L-8937F	L	Forward	TTCTTCTGAGATTGAGCGGC
YEZV_L-9572F	L	Forward	GAGGCCTGATGATGGACAAG
YEZV_L-9713R	L	Reverse	TTGGCACTTGTGGAGACTTC

Primer name	Target segment	Direction	Sequence (5' -> 3')
YEZV_L-10443R	L	Reverse	GGTTGGAAGCAGGTTGAGAA
YEZV_L-11006F	L	Forward	CCCCGAATCTGCAGCTAAAT
YEZV_L-11348R	L	Reverse	GCTGACAATTCGTTCTTGGC
YEZV_L-11586R	L	Reverse	AGAAGCATCCTTCCTAAGCG
YEZV_M-222F	M	Forward	TGACACCAGGAAACACGACC
YEZV_M-437F	M	Forward	CTGATCAGGGCAGTCGATCC
YEZV_M-1289R	M	Reverse	AACAGCCCTTGACTCCACTG
YEZV_M-1377R	M	Reverse	TTGTCCACCAGGCATTTACC
YEZV_M-1675F	M	Forward	GGTAGTTTTCGGCTACAGCA
YEZV_M-1699R	M	Reverse	ACCAATGCTGTAGCCGAAAA
YEZV_M-2508F	M	Forward	GAAACGTCTTTGTCTCGATCCTTG
YEZV_M-2570R	M	Reverse	TCAGGTATTCGAACTGAGTTTCGT
YEZV_M-2531F	M	Forward	GAGTATGCTCAGGAGTACGAAACT
YEZV_M-2908R	M	Reverse	ACTGAAAGAGACTGATATGCCTCC
YEZV_M-3387F	M	Forward	TAACAGCCCTTCTGAGCGTG
YEZV_M-3483R	M	Reverse	TAACAGCCTTCGCACATGGT
YEZV_M-3543R	M	Reverse	TACCGGTCAGGACTGTCCAA
YEZV_M-3605F	M	Forward	AGCGCTGGACAAGTGAATGA
YEZV_M-3817R	M	Reverse	AGACAAGAACCCTTTCCCGC
YEZV_S-116F	S	Forward	CCAAGTACACGAAATCCCTTGCTCT
YEZV_S-269R	S	Reverse	TCTGGGATTGGAGCAGCAAA
YEZV_S-389F	S	Forward	CATCTCCCGAGGATGTTGCA
YEZV_S-717F	S	Forward	AAAGAAGGGCCTGCTGCTAG
YEZV_S-928R	S	Reverse	TGATTAGCCCCTCTGCCTCT
YEZV_S-1233R	S	Reverse	GGCTGCAGTCATCTCAGACA
YEZV_S-1210F	S	Forward	AGAATGTCTGAGATGACTGCAG
YEZV_S-1434R	S	Reverse	AGACTGATGGAAGTAGTGCTCAGGT

Supplementary Table 4. Primers used for terminal sequencing

Primer name	Segment	Sequence (5' -> 3')
YEZV_L-64R	L	TGGAAAGCTTGCCTGCCATCA
YEZV_L-89R	L	TGCTCCCATGTCAACTCAGGC
YEZV_L-178R	L	AGAATCTCAGCCTCAATTGAACCT
YEZV_L-11685F	L	AGCAACTTTGGTGGGCCGAA
YEZV_L-11769F	L	TGCCACAGAAGACGACCTCCT
YEZV-M-131R	M	TGTTCAGAGACCCATCGCCG
YEZV-M-195R	M	TTTGTGGGTGCTACTGCGCT
YEZV_M-3969F	M	GCCTCATCAAGGTCTGCATCAGT
YEZV_M-4080F	M	AGGAACTGCTGAGGAGGAACGA
YEZV_S-48R	S	TCAGACGTGCCATCTCCGGT
YEZV_S-174R	S	GGCAGGAGCCAGGTTCACAT
YEZV_S-1495F	S	CCCTGCGTGTCAACATCGTG
YEZV_S-1526F	S	GGCCGGGGGAACCCATTAAA
YEZV_S-584R	S	CCTGTGCCTTCTCTTGCTCCTCATGTC
YEZV_S-972F	S	TCAGCCCTTGACACTGCATTTTC
DT88	Linker	/PHO*/GAAGAGAAGGTGGAAATGGCGTTTTGG/PHO/
DT89	Linker	CCAAAACGCCATTTCCACCTTCTCTTC

*phosphorylation

Supplementary Figures

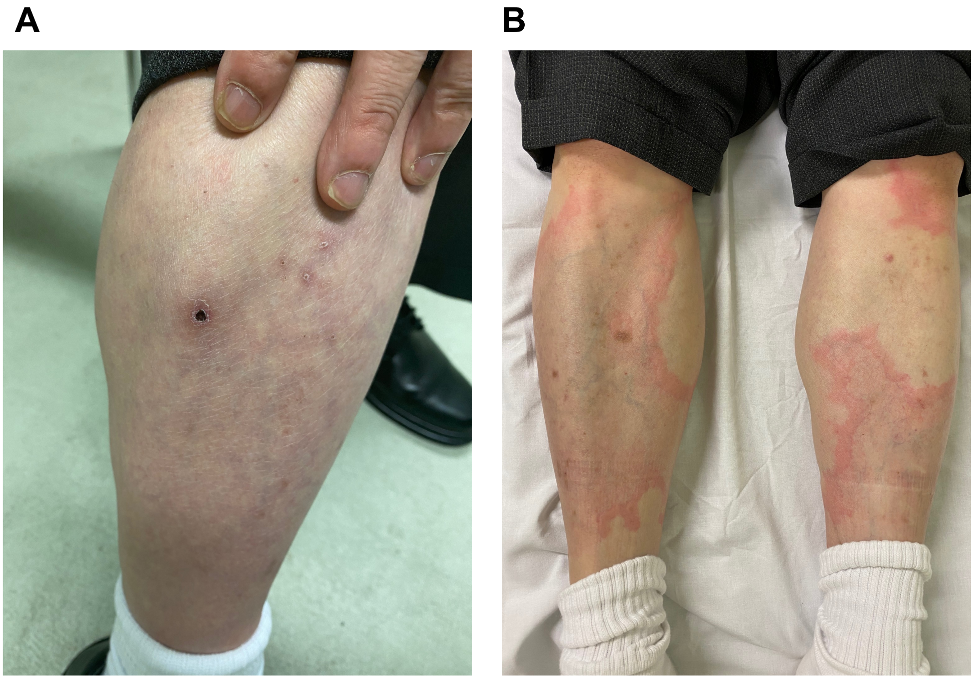

Supplementary Figure 1. Skin lesions exhibited by patient 2.

A) A small eschar lesion following attachment of an arthropod on the lateral aspect of the right lower extremity (day 5 after the onset of the fever). B) Pruritic urticarial rash on bilateral lower extremities (day 6).

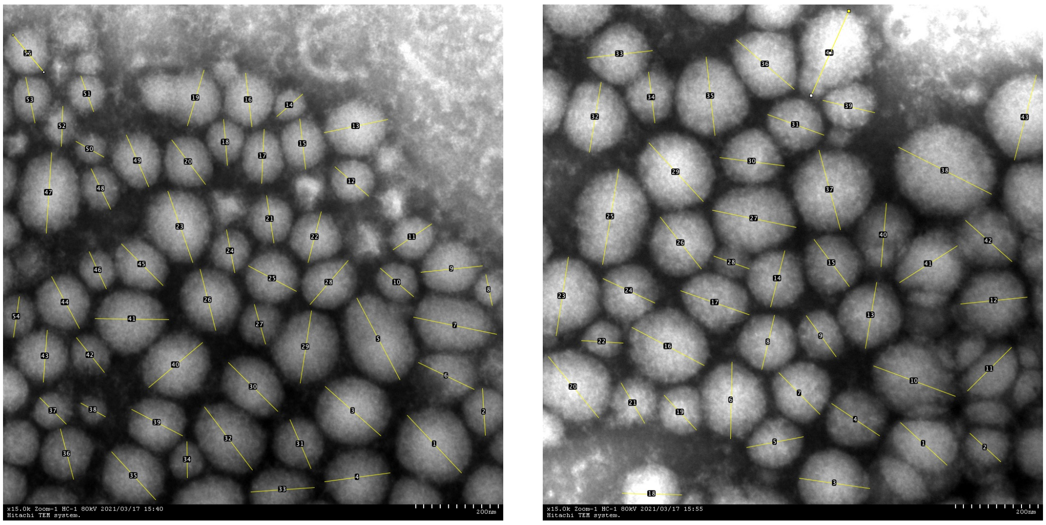

Supplementary Figure 2. Measurement of long diameters of Yezo virus particles

Long diameters of virus particles captured using a transmission electron microscope were labeled manually (yellow lines). Line length was measured using the scales in the images with Fiji software.
